# Combining Polygenic Hazard Score With Volumetric MRI and Cognitive Measures Improves Prediction of Progression From Mild Cognitive Impairment to Alzheimer's Disease

**DOI:** 10.3389/fnins.2018.00260

**Published:** 2018-04-30

**Authors:** Karolina Kauppi, Chun Chieh Fan, Linda K. McEvoy, Dominic Holland, Chin Hong Tan, Chi-Hua Chen, Ole A. Andreassen, Rahul S. Desikan, Anders M. Dale, The Alzheimer's Disease Neuroimaging Initiative

**Affiliations:** ^1^Department of Radiology, University of California, San Diego, La Jolla, CA, United States; ^2^Department of Radiation Sciences, University of Umea, Umea, Sweden; ^3^Department of Cognitive Sciences, University of California, San Diego, La Jolla, CA, United States; ^4^Department of Neurosciences, University of California, San Diego, La Jolla, CA, United States; ^5^Neuroradiology Section, Department of Radiology and Biomedical Imaging, University of California, San Francisco, San Francisco, CA, United States; ^6^NORMENT, Institute of Clinical Medicine, Division of Mental Health and Addiction, University of Oslo, Oslo University Hospital, Oslo, Norway; ^7^Division of Mental Health and Addiction, Oslo University Hospital, Oslo, Norway

**Keywords:** pHs, MCI, AD prediction, MRI, genetics

## Abstract

Improved prediction of progression to Alzheimer's Disease (AD) among older individuals with mild cognitive impairment (MCI) is of high clinical and societal importance. We recently developed a polygenic hazard score (PHS) that predicted age of AD onset above and beyond *APOE*. Here, we used data from the Alzheimer's Disease Neuroimaging Initiative (ADNI) to further explore the potential clinical utility of PHS for predicting AD development in older adults with MCI. We examined the predictive value of PHS alone and in combination with baseline structural magnetic resonance imaging (MRI) data on performance on the Mini-Mental State Exam (MMSE). In survival analyses, PHS significantly predicted time to progression from MCI to AD over 120 months (*p* = 1.07e-5), and PHS was significantly more predictive than *APOE* alone (*p* = 0.015). Combining PHS with baseline brain atrophy score and/or MMSE score significantly improved prediction compared to models without PHS (three-factor model *p* = 4.28e-17). Prediction model accuracies, sensitivities and area under the curve were also improved by including PHS in the model, compared to only using atrophy score and MMSE. Further, using linear mixed-effect modeling, PHS improved the prediction of change in the Clinical Dementia Rating—Sum of Boxes (CDR-SB) score and MMSE over 36 months in patients with MCI at baseline, beyond both *APOE* and baseline levels of brain atrophy. These results illustrate the potential clinical utility of PHS for assessment of risk for AD progression among individuals with MCI both alone, or in conjunction with clinical measures of prodromal disease including measures of cognitive function and regional brain atrophy.

## Introduction

Late onset Alzheimer's disease (AD) is the most common form of dementia, affecting 24–35 million people world-wide (Querfurth and LaFerla, 2010; Alzheimer's Association, 2015). Novel methods to enable early AD detection based on clinically feasible, economical, and non-invasive measures is of high clinical and societal value. Early identification of high-risk individuals is also of utmost importance for pre-dementia clinical trials (Holland et al., 2012; Ritchie et al., 2016).

A large effort has been made to improve the prediction of progression to AD among older individuals with mild cognitive impairment (MCI), which can be a transition stage from normal age-related cognitive decline to dementia (Roberts and Knopman, 2013). Next to older age, inheritance of the ε*4* allele of the *Apolipoprotein E* (*APOE)* gene is the strongest individual risk factor for late onset AD (Yu et al., 2014). However, other genetic risk variants of smaller effect also contribute to AD risk (Lambert et al., 2013; Ridge et al., 2013). While most studies examining genetic risk for sporadic AD focus on *APOE* genotype, some have assessed polygenic risk scores beyond *APOE* based on case-control data from large-scale genome-wide association studies (GWAS) (Lambert et al., 2013), and found that genetic variants associated with elevated AD risk also influence brain structure (Sabuncu et al., 2012) and cognitive function (Marioni et al., 2017). Given that the incidence of AD increases sharply with age, we recently develop a polygenic hazard score (PHS) for prediction of age-specific AD risk, based on 31 AD-susceptibility variants, including *APOE* (Desikan et al., 2017). The PHS showed substantial improvement over *APOE* in predicting age of AD onset and was associated with biomarkers of AD, including MRI-based hippocampal volume loss (Desikan et al., 2017), amyloid, and tau deposition (Tan et al., 2018).

Volumetric MRI-based measures of regional brain atrophy, particularly medial temporal volume loss are important biomarkers for assessing risk of progression to AD in patients with MCI (Li et al., 2016). In previous work from our lab, we derived a composite regional “brain atrophy score” from linear discrimination analysis trained on data from healthy controls and AD patients, which was better at predicting 1 year cognitive decline than atrophy in medial temporal structures alone (McEvoy et al., 2009). The atrophy score is based on volume of the hippocampus, and thickness of entorhinal cortex, middle temporal gyrus, bank of the superior temporal sulcus, isthmus cingulate (retrosplenial cortex), superior temporal gyrus, medial and lateral orbitofrontal gyri, with weightings for each ROI determined through a linear discrimination analysis that best distinguished AD patients from healthy controls. In a subsequent study, this brain atrophy score predicted 1-year risk of progression to AD in individual patients (McEvoy et al., 2011). Prediction of 1-year clinical decline was further improved by adding subjects' baseline Mini-Mental State Exam (MMSE) scores and number of *APOE* ε4 alleles to the model (McEvoy et al., 2009).

In the current study, we investigated the clinical utility of PHS for individual assessment of risk for clinical progression to AD over time among older individuals with MCI, a critical question for most patients admitted to memory clinics. Whereas prior work from our group has shown the value of PHS for predicting AD-associated clinical and cognitive decline among non-demented elderly individuals (Tan et al., 2017), a critical next step in assessing the potential clinical utility of PHS is to examine the extent to which PHS provides independent information beyond other commonly used predictors, such as brain atrophy levels and baseline cognitive function, and to determine whether combinations of these measures improve prediction of clinical decline and progression to dementia. To this end, we used survival analyses (Klein et al., 2013) to compare single-, two-, and three-factor models of PHS, atrophy score, and MMSE for prediction of time to progression from MCI to AD over 120 months of follow-up. As a complementary approach, we used linear mixed-effect modeling to examine prediction of clinical change in MMSE and the Clinical Dementia Rating, sum of boxes (CDR-SB), over 36 months.

## Methods

### Participants

We used participants from the ADNI database, available as of November 2011. ADNI 1 is a 5-year multi-site program launched in 2003 as a public-private partnership including the National Institute on Aging, Food and Drug Administration, pharmaceutical companies, and nonprofit organizations (led by Principal Investigator Michael W. Weiner, MD.). The main goal of ADNI is to examine if progression from MCI to AD can be predicted based on neuroimaging, biological biomarkers as well as clinical and neuropsychological assessments.

The baseline data was collected in 2005, including elderly healthy controls (*n* = 200), Alzheimer's disease patients (*n* = 200), and individuals with MCI (*n* = 400), followed by annual follow-ups for 36 months. We included 336 participant with a MCI diagnosis at baseline in ADNI 1 and available genetic, MRI, and cognitive data, including mini-mental state examination (MMSE) (Folstein et al., 1975) and Clinical dementia rating, sum of boxes (CDR-SB) (Hughes et al., 1982). The age range was 55–89 at baseline. Longitudinal data on CDR-SB and MMSE was included from ADNI 1, with 36-month follow-up. Data on progression to AD was also included from ADNI 2 and ADNI GO, providing data on progression to AD for up to 120 months after baseline. The ADNI study was approved by local institutional review boards, and all participants or participant's guardians provided written informed consent. Additional information about ADNI is available at http://www.adni-info.org.

### MRI acquisition and analyses

Details of image acquisition and analysis have been described in our previous publications (McEvoy et al., 2009). Briefly, we downloaded the raw baseline DICOM MRI data from the ADNI web site (http://adni.loni.usc.edu/data-samples/mri/) and obtained volumetric assessments on neuroanatomic regions of interest (ROIs) using a modified version of the FreeSurfer image-analysis software (Brewer, 2009; Brewer et al., 2009). We used a previously validated brain atrophy score based on volume of the hippocampus, and thickness of entorhinal cortex, middle temporal gyrus, bank of the superior temporal sulcus, isthmus cingulate (retrosplenial cortex), superior temporal gyrus, medial and lateral orbitofrontal gyri, with weightings for each ROI determined through a linear discrimination analysis that best distinguished AD patients from healthy controls (for additional details, see McEvoy et al., 2009, 2011). In the current paper, we computed an atrophy score as the sum of weighted measures from these brain regions, averaging left, and right hemispheres.

### Polygenic hazard score (PHS)

For each participant included in the study, we calculated their individual PHS based on our previous publication (Desikan et al., 2017). In brief, PHS was derived by first identifying common variants associated with AD in the International genetics of Alzheimer's Project (IGAP) GWAS on AD, with a *p*-value threshold of <10^−5^ using summary statistics. These SNPs were examined for association with AD in the Alzheimer's Disease Genetic consortia (ADGC) phase 1 genetic data. A stepwise Cox proportional hazard model was applied in which the SNPs that improved the model most were included sequentially until the model was no longer improved by adding more SNPs. This resulted in a list of 31 SNPs, including the two SNPs that constitute the *APOE* ε genotype, which were used to generate the PHS. Finally, the PHS estimates from ADGC were integrated with established AD-incidence rates from the US population to provide quantitative estimates of the annualized (cumulative) incidence rate. The PHS is the vector product of a person's genotype for the 31 SNPs and the corresponding parameter estimates from the Cox proportional hazard model.

### Statistical analyses

We used Cox proportional Hazard models in Matlab [version 8.5.0.197613 (R2015a)] to model time to progression from MCI to AD with a follow up period of 120 months. Time to event was defined as time from baseline to AD onset (with end of study time or drop out as censoring). We used Kaplan–Meier survival analysis to determine the time to progression for the 10th, 50th, and 90th risk percentile. We first fitted a baseline model containing age, age^2^, sex, and age^*^sex as predictors. We then added PHS to the baseline model, and used log likelihood ratio to assess whether PHS significantly improved the prediction. Model comparisons between PHS and number of *APOE* ε4 alleles were also made (Baseline and *APOE* vs. Baseline, *APOE* and PHS). Thereafter, we examined the combined model of PHS and atrophy score at baseline, and performed model comparisons with each factor alone. Finally, we added baseline measure of MMSE to a three-factor model, and performed model comparisons among all two-factor models. Cross-validated prediction accuracy, sensitivity, specificity and area under the curve (AUC) were calculated via the receiver operator characteristics (ROC) analyses using the perfcurve function in Matlab (MathWorks). The assumption of proportional hazards was not violated for any of the included covariates (*p*'s > 0.05, as evaluated by scaled Schoenfeld residuals using the cox.zph function in R).

To predict cognitive decline in patients with MCI, we used linear mixed-effects models to estimate change in MMSE or CDR-SB over 36 months. Mixed effects models were fitted via maximum likelihood by using the lmer function in R (version 3.2.3). Sex, age, education, and five genetic principal components to control for population stratification were included in all analyses as baseline variables. We included PHS, atrophy score and the combination of PHS and atrophy score in three different models each for prediction of change in CDR-SB and MMSE. The models allowed for random subject-specific intercept and slope. Model comparisons of *APOE* and *APOE*+PHS were performed, where *APOE* denotes the number of *APOE* ε4 alleles. Likelihood ratio test via ANOVA were used for all model comparisons.

## Results

Of the 336 participants with MCI at baseline, 182 developed AD within the follow-up period of 120 months. Baseline demographics of stable MCI (MCI-s) and MCI patients that subsequently progressed to AD (MCI-c) are presented in Table 1. The groups did not differ in sex distribution, age, or education. As expected, *APOE* ε4 alleles, MMSE, atrophy score, and PHS were related to subsequent progression.

**Table 1:** Clinical demographics.

	**MCIs (*n* = 154)**	**MCIc (*n* = 182)**	**Statistics**	***p***
Males, *n* (%)	99 (64%)	117 (64%)	$\chi_{\left(1\right)}^{2}$ = 0	1
Age, y (*SD*)	75.84 (7.4)	74.92 (6.9)	*t*_(334)_ = −1.18	0.24
APOE4 +, *n* (% E4+)	69 (45%)	117 (64%)	$\chi_{\left(1\right)}^{2}$ = 12,	5.2e^−4^
Education	15.68	15.82	*t*_(334)_ = 0.41	0.68
MMSE	27.37	26.87	*t*_(333)_ = −2.59	0.01
Atrophy score	2382	2207	*t*_(330)_ = −4.45	1.17e^−5^
PHS	0.356	0.661	*t*_(334)_ = 3.54	4.42e^−4^

*Clinical and demographics data at baseline for patients with stable MCI (MCIs) and those who converted to AD within the study period (MCIc). MCI, mild cognitive impairment; PHS, Polygenic hazard score; SD, standard deviation*.

**Figure 1:**
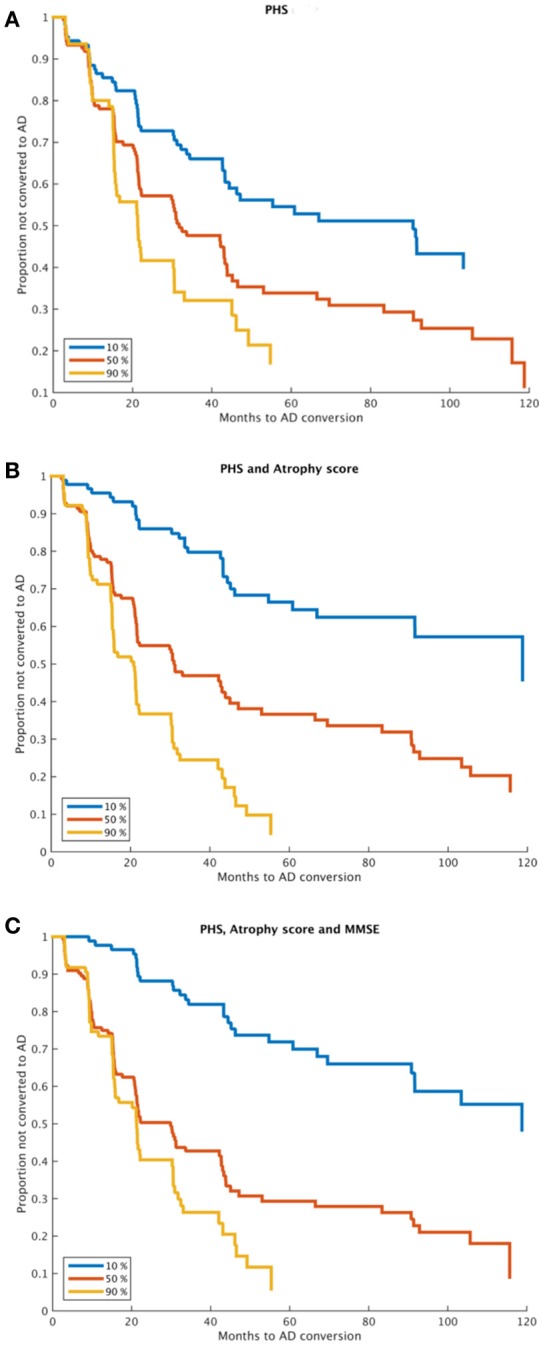
Time to MCI to AD progression. Kaplan-Meier survival curves for 10, 50, and 90th percentiles of **(A)** PHS, **(B)** PHS and atrophy score, and **(C)** PHS, atrophy score and MMSE (at baseline). Model comparisons using log likelihood of the two-factor model **(B)** vs. any one-factor model, and the three-factor model **(C)** vs. any combination of two-factor models; all *p* < 0.005. PHS, Polygenic Hazard Score; AD, Alzheimer's Disease.

Estimated survival functions for the 10th, 50th, and 90th percentile based on one-, two-, and three-factors cox proportional hazard models modeling time to progression from MCI to AD are shown in Figure 1 (Figure 1A, PHS, Figure 1B, PHS and atrophy score, Figure 1C, PHS, atrophy score, and MMSE), and model summaries are shown in Table 2. The PHS significantly predicted progression from MCI to AD over 120 months follow-up (*p* = 1.07e-5), and PHS was a significantly stronger predictor of progression than *APOE* ε genotype (*p* = 0.0152, for model comparison of *APOE* vs. *APOE* +PHS). When including atrophy score (McEvoy et al., 2009) in the model, PHS remained significant and the two-factor prediction model was significantly more predictive than either single-factor model (*p*'s = 5.61e-11, and 0.0015 for comparison with single-factor models of PHS and atrophy score, respectively). Finally, we included cognitive functioning at baseline (MMSE) to a three-factor prediction model, which yielded a combined model *p*-value of 4.28e-17. Model comparisons showed that the three-factor model was significantly more predictive than the two-factor model (*p* < 0.005).

**Table 2:** Results from Cox model analyses.

**Variables**	**Log(HR)**	**SE**	***t***	***p***
**BASELINE VARIABLES**				
Age	−0.229	0.192	−1.190	0.234
Age^∧^2	0.001	0.001	0.907	0.364
Sex	−2.788	1.706	−1.634	0.102
Sex^*^age	0.035	0.022	1.554	0.120
**SINGLE-FACTOR MODELS**				
PHS	0.454	0.097	4.684	2.81E-06
Atrophy score	−0.002	2.45E-04	−7.276	3.44E-13
MMSE	−0.205	0.044	−4.629	3.68E-06
**TWO-FACTOR MODEL**				
PHS	0.314	0.099	3.175	0.001
Atrophy score	−0.002	2.54E-04	−6.463	1.03E-10
**THREE-FACTOR MODEL**				
PHS	0.264	0.100	2.644	0.008
Atrophy score	−0.002	2.58E-04	−5.962	2.49E-09
MMSE	−0.142	0.047	−3.010	0.003

*Baseline variables incorporated in all subsequent models. PHS, Polygenic hazard score; MMSE, mini-mental state examination; HR, Hazard ratio*.

ROC analyses were performed to assess performance accuracies for one-, two- and three-factor models for prediction of progression over a 36 months follow-up period based on combinations of atrophy score, MMSE, and PHS (Table 3). In brief, the atrophy score had an accuracy of 74.6, sensitivity of 77.8, and a specificity of 70.8. Adding PHS to the atrophy score increased specificity, at the cost of sensitivity; with increased overall accuracy and AUC (although confidence intervals overlapped). Similar results were seen for comparisons of MMSE alone and in combination with PHS. The full three-factor model had the highest AUC (.84), accuracy (78.9), sensitivity (79.9), and a specificity of 77.8.

**Table 3:** Prediction accuracy.

**Variables**	**Accuracy (%)**	**Sensitivity (%)**	**Specificity (%)**	**AUC (95% CI)**
**PREDICTORS**				
MMSE	68.4	62.8	75.1	0.73 (0.68–0.78)
Atrophy score	74.6	77.8	70.8	0.79 (0.74–0.83)
PHS and MMSE	70.0	57.2	85.1	0.79 (0.74–0.83)
PHS and atrophy score	76.1	72.9	79.8	0.82 (0.77–0.86)
MMSE and Atrophy score	77.2	77.08	77.3	0.82 (0.77–0.86)
Atrophy score, PHS and MMSE	78.9	79.9	77.8	0.84 (0.79–0.88)

*Prediction of progression from MCI to AD by 36 months follow up. PHS, polygenic hazard score; MMSE, mini-mental state examination; MCI, mild cognitive impairment; AD, Alzheimer's Disease; AUC, area under the curve*.

Results from the linear mixed-effect models for prediction of change in MMSE and CDR-SB over 36 months are presented in Table 4. Model comparisons showed that PHS significantly improved prediction of both MMSE (χ^2^ = 26.7, df = 1, *p* = 2.34e-07) and CDR-SB (χ^2^ = 21.57, df = 1, *p* = 3.41e-06) compared to the baseline variables. Further, the PHS performed significantly better than *APOE* ε4 status in prediction of both MMSE (χ^2^ = 8.61, df = 1, *p* = 0.0033) and CDR-SB (χ^2^ = 6.12, df = 1, *p* = 0.013). Again, PHS remained significant after adding atrophy score to the model (Table 4). Compared to atrophy score alone, the combined model of PHS and atrophy score was significantly more predictive of change in both MMSE (χ^2^ = 19.04, df = 1, *p* = 1.281e-05, [controlling for *APOE* ε4 alleles: χ^2^ = 6.97, df = 1, p = 0.008]) as well as CDR_SB (χ^2^ = 13.43, df = 1, *p* = 0.00025 [controlling for APOE ε4 alleles: χ^2^ = 4.57, df = 1, *p* = 0.033]).

**Table 4:** Prediction of clinical decline.

**Model**	**Predictors**	**Estimate**	**SE**	***t***	***p***
**OUTCOME: MMSE**					
PHS	PHS	−0.87	0.17	−5.2(253.2)	4.06e−07
PHS+ Atrophy score	PHS	−0.72	0.17	−4.34 (251.5)	2.05e−05
	Atrophy score	2.51	0.67	3.73 (243.6)	0.000238
**OUTCOME: CDR-SB**					
PHS	PHS	0.46	0.10	4.65(259.3)	5.28e−06
PHS+ Atrophy score	PHS	0.42	0.10	4.27 (256.1)	2.80e−05
	Atrophy score	−1.79	0.38	4.70 (249.3)	4.39e-06

*Effect of predictors on change in outcome measure over 36 months. PHS, Polygenic Hazard score; MMSE, mini mental state examination; CDR-SB, Clinical Dementia Rating score, sub of Boxes*.

## Discussion

Using the ADNI dataset, we assessed the potential clinical utility of the recently established age-specific AD PHS (Desikan et al., 2017) to more accurately predict the progression to AD among patients diagnosed with MCI. Examining both time to AD diagnosis and decline in clinical scores (CDR-SB and MMSE), we found that PHS significantly predicted clinical progression to AD beyond *APOE* genotype. Critically, PHS remained a significant predictor even when regional brain atrophy levels and cognitive functioning was known, and the prediction models were significantly improved by adding PHS to models containing atrophy score and/or MMSE. These results show that the predictive value of PHS is, at least partly, independent of brain atrophy and cognitive functioning at the MCI stage. In a typical memory clinic setting, PHS may be used to assess AD risk before other diagnostics have been performed, and may also be valuable for further improvement of prediction after an individual has underwent an MRI examination.

We used two complementary approaches to assess clinical progression among MCI patients. First, we used survival analyses to assess time to progression from MCI to AD over a long period of 120 months, using combinations of PHS, MRI, and cognitive functioning (Figure 1). The PHS was a significant predictor both alone and when controlling for atrophy level and cognitive function at study start, and model comparisons showed significant model improvements when adding PHS to models consisting of atrophy score and/or MMSE. ROC analyses showed that adding PHS to atrophy score or MMSE improved prediction accuracy, which was primarily driven by increased specificity, but at the cost of lower sensitivity (Table 3). These results suggest that different combinations of biomarkers may be used in different clinical situations where higher sensitivity or specificity is prioritized. As expected, the best model performance was derived from the full three- factor model with PHS, MRI, and cognitive assessment.

Secondly, we used linear mixed-effect models to examine the influence of PHS on change in CDR-SB and MMSE over a shorter time period, 3 years, in the same individuals with MCI at baseline. In line with the results from survival analyses, PHS significantly predicted clinical decline of both CDR-SB and MMSE among elderly diagnosed with MCI beyond *APOE* status, both individually and when atrophy levels were included in the model (Table 4). Taken together, these results show the utility of the PHS to assess individual risk for clinical decline in patients diagnosed with MCI also when their current levels of brain atrophy and cognitive functioning is known.

Previous studies using both survival analyses and linear mixed effect models showed improved prediction of AD progression by combining MRI data with CSF biomarkers (Vemuri et al., 2009; Westman et al., 2012), *APOE* genotype (McEvoy et al., 2009; Dukart et al., 2015), and different cognitive test batteries (Callahan et al., 2015; Eckerström et al., 2015; Li et al., 2016). Investigators from our group have shown that the combination of cognitive performance and medial temporal atrophy substantially improves prediction of MCI to AD progression in comparison to prediction based on individual risk factors (Heister et al., 2011), and also that the combination of *APOE* genotype and brain atrophy outperforms models based on either variable alone (McEvoy et al., 2009). Here, we extend previous models based on *APOE* to our recently developed PHS based on whole-genome data. It is still not fully known through which mechanisms *APOE* and other genes with smaller effect impact AD risk, but brain atrophy level and cognitive function are considered intermediate phenotypes that may mediate genetic effects on AD risk. In our previous paper, we found a correlation between PHS and larger volume loss in AD-related brain areas (Desikan et al., 2017). The current findings that PHS is predictive of AD progression when levels of brain atrophy are included in the model, shows that MRI biomarkers are not fully mediating the effect of PHS. Prediction based on genetic testing has the advantages of being relatively cheap, non-invasive, and not time-sensitive (since genetic assessment only has to be carried out once, is valid for a whole life time and can be used for multiple clinical purposes). In contrast to polygenic risk scores developed in a case/control framework, PHS is focused on predicting age of onset, which more accurately captures the increase in population incidence with increased age, where older age is the strongest risk factor for AD development.

## Limitations

ADNI is one of the largest longitudinal dataset for studying progression from MCI to AD, but has limitations. First, participants were recruited from memory clinics and advertisements, and MCI inclusion criteria were highly selective, thus the study group is not representative of the general population. Further, AD diagnosis has not been confirmed with histopathology. Also, study dropouts are biased toward high-risk individuals, which might lead to a bias in the estimates of progression rates. As high-risk individuals are more likely to have a higher PHS, the predictive value of the PHS might be underestimated in this study.

## Conclusions

The present study shows that the prediction of clinical progression to AD among MCI patients can be improved by combining the age-sensitive PHS with structural neuroimaging and baseline cognitive ability. Improved individual assessment of AD risk among elderly patients presenting with subjective memory complaints could be helpful in clinical practice to determine treatment plans, and is also of high importance for intervention studies where recruitment of high-risk individuals at an early stage of the disease process is crucial for testing effectiveness of new disease-altering interventions.

## Ethics statement

The ADNI study was approved by the local ethics committee at each center. All subjects gave written informed consent in accordance with the Declaration of Helsinki. For more information see ADNI webpage; http://adni.loni.usc.edu.

## Author contributions

KK, CF, RD, and AD: designed the study; CF and KK: performed analyses; KK: wrote the manuscript; CF, LM, DH, CT, C-HC, OA, RD, and AD: read and provided comments on the manuscript.

### Conflict of interest statement

LM holds equity in CorTechs Labs, Inc. AD is a founder of and holds equity in CorTechs Labs, Inc., and serves on its Scientific Advisory Board. He is also a member of the Scientific Advisory Board of Human Longevity, Inc. (HLI), and receives research funding from General Electric Healthcare (GEHC). The terms of these arrangements have been reviewed and approved by the University of California, San Diego in accordance with its conflict of interest policies. OA has a patent SYSTEMS AND METHODS FOR IDENTIFYING POLYMORPHISMS pending. The other authors declare that the research was conducted in the absence of any commercial or financial relationships that could be construed as a potential conflict of interest.
